# It’s not evidence, it’s insight: bringing patients’ perspectives into health technology appraisal at NICE

**DOI:** 10.1186/s40900-016-0018-y

**Published:** 2016-03-24

**Authors:** Kristina Staley, Caroline Doherty

**Affiliations:** TwoCan Associates, 45 Portland Road, Hove, BN3 5DQ UK

**Keywords:** Health technology assessment, Health technology appraisal, Health technology assessment committees, Patient and public involvement, Public involvement, Evidence

## Abstract

**Plain English summary:**

Health technology appraisal involves reviewing the findings from clinical trials and economic data to produce guidance on how health technology should be used. This task is carried out by appraisal committees in NICE. One of the several ways in which patients can feed their views into these committees is via a written patient statement. We asked nine committee members about what difference the information from patients makes to their decision-making. The Committee members reported that written patient statements offer a different perspective when reviewing the clinical and economic data. This can have a profound impact when a committee draws conclusions based solely on the data, which may not reflect the reality of patients’ lives. The patients’ and carers’ input provides meaning to the data, ‘bringing the numbers to life’. It identifies if the technology has any wider impacts than what’s been reported in the clinical trial, and also if the trial has measured what’s important to patients. We conclude that the written patient statement adds value to the decision-making process by helping Committee members to make sense of the clinical and economic data-it makes them look at the evidence ‘in a different light’. Patients’ stories are very effective in this context, because they have the power to communicate and to challenge Committee members’ assumptions. Understanding this difference between analysing research evidence and drawing on patients’ insights is important in thinking about what’s needed in a written patient statement and the best way to obtain it.

**Abstract:**

**Background**

Health technology appraisal involves reviewing clinical and economic data to inform guidance on the use of technology. In England this task is carried out by appraisal committees within the National Institute for Health and Care Excellence (NICE). Patients are not committee members as they have a vested interest in the outcome, but one of the several ways they are involved is through submitting a written patient statement, which is considered by the committee during its deliberations. We aimed to find out how the written patient statement adds value to the decision-making process by exploring how it is used in practice.

**Methods**

Semi-structured interviews were conducted with nine members of NICE appraisal committees. The interviews were transcribed and analysed thematically. We drew on published evidence of the impact of patient involvement on clinical research and our experience of supporting organisations to produce written patient statements to analyse the findings.

**Results**

Committee members reported that written patient statements offer a different perspective when evaluating clinical and economic data. This can have a profound impact when a committee draws conclusions based on data that may not reflect the reality of the patient experience. Information from patients and carers also provides context and meaning to the data, by explaining its real-life implications. It identifies wider impacts of a technology that may not have been assessed in a clinical trial, as well as commenting on whether what has been measured in a trial is relevant to patients. The main barrier to using the written patient statement is the misperception that it is a form of research ‘evidence’, when in fact it takes the form of experiential knowledge - or insight.

**Conclusions**

The written patient statement adds value by aiding Committee members in their interpretation of existing evidence – it enables them to consider this evidence ‘in a different light’. In this context, patients’ experiential knowledge is effective because it is subjective, emotional and anecdotal. It then has the power to communicate and to challenge assumptions based on the data alone. Understanding this difference between using evidence and insights has implications for the content of a written patient statement and the approaches used to obtain it.

## Background

Health technology appraisal is a multi-stage process with potential for patient and public involvement at all stages. The entire process includes: setting priorities for appraisal, scoping the questions to be addressed, carrying out an economic analysis, evaluation of the clinical and economic research evidence (technology appraisal) and communication of the final outcome [1–5]. In this article, we consider the value of patient and public involvement in technology appraisal, where consideration is given to clinical and economic data as well as wider legal, social and ethical issues, to inform guidance on the use of new and existing technologies. In England this task is carried out by appraisal committees within the National Institute for Health and Care Excellence (NICE) [6].

Within NICE, the views of patients, carers and the public are included in the appraisal process in a number of ways. At the initial scoping stage, interested patient organisations and charities participate in a consultation to help set the questions to be answered during the appraisal. They can also register as stakeholders when the guidance development starts, which enables them to be involved in later stages as described below.

At the stage of evaluating the research evidence, there are two distinct roles for the public and/or patients. One is as a member of an appraisal committee, i.e., someone who participates in the process. This is the role of the lay members of NICE committees. Importantly these lay members do not include patients with direct experience of the condition under review. It can be argued that such patients should not be party to the decision-making process, because they have a vested-interest in the outcome. For the same reason, representatives of the manufacturers of the technology being assessed are not included as committee members.

However, it is recognised that because any decision will have a major impact on patients’ lives, patients and carers do have a right to have their views heard by the committee. Therefore, the registered patient groups and organisations are invited to produce a ‘patient statement’, a written report, which is considered at the same time as the clinical data produced by the manufacturers and economic data from health economists [7]. Expert patients and/or their representatives from the registered stakeholder organisations are selected by NICE to attend part of the appraisal committee meeting and can make comments on the day. They usually include someone from a patient organisation and someone with experience of the condition and the technology under review. The expert patients/ representatives also produce a written summary of their experiences, which is included in the meeting papers.

It has been argued that there is a need to seek patients’ views during the appraisal, because patients can provide a real-world view of the social and psychological impacts of the use of a technology [2, 3]. They can also provide an understanding of the ‘burden of their illness’ and their experience and expectations of current treatments. However, there is no clarity or transparency as to how such information influences a committee’s deliberative process. Although templates have been produced for written patient statements, it is still unclear as to what precise information is most useful to include and the questions asked can seem very broad. The main conclusions to date are that this ‘evidence’ should be obtained through qualitative research conducted by social scientists [2, 3]. On this basis, it has also been suggested that appraisal committees should include professionals with experience in social or humanistic sciences, to ensure rigorous methodological analysis and an evaluation of the quality of this patient evidence, in much the same way that clinicians and economists assess the clinical and economic data [2].

In this article, we address the question: How do the views of patients directly affected by a new technology add value to the appraisal process? We interviewed academic, clinical and lay members of appraisal committees to ask how they had used the written statements from patient organisations and how this had influenced their decision-making in practice. We have also drawn on the literature describing the impact of patient and public involvement on clinical research and our own experience of working with patient groups to produce written patient statements for the former NHS National Specialised Commissioning Team (NSCT). We have analysed these findings to develop a clearer understanding of the kind of information from patients that can best support and enhance decision-making within appraisal committees.

## Methods

### Interviews with appraisal committee members

Semi-structured telephone interviews lasting 30–45 mins, were conducted with nine members of NICE health technology appraisal committees in England, during February and March 2014. We approached all thirteen members of a single committee (which has not been identified to protect the anonymity of the interviewees). Only nine were available to take part in the time available. Some had been members of other appraisal committees in NICE and were able to draw on several years of experience. They came from a range of disciplines including consultants from different areas of medicine, health service managers, lay members (from patient organisations and business) and university academics working in health service research.

As NICE had only commissioned us to explore the use of written patient statements, we focused our questions on whether and how these statements made a difference to the committees’ decision-making and how the quality of this information might be improved. With the interviewee’s permission, the interviews were recorded and transcribed. The transcripts were analysed using inductive thematic analysis [8, 9], i.e., the themes were developed from the interview data, rather than drawing on an existing theory. The initial development of themes was carried out by KS, and discussed with CD to develop the conclusions. We drew on our experience of public involvement in research and experience of producing written patient statements to inform our analysis. Five distinct themes emerged. Four related to the added-value of the written patient statement, in terms of aiding understanding of the health condition under review, the benefits and risks of the new technology, the wider impacts of the new technology and the relevance of the clinical research. The fifth theme related to barriers to the use of the written patient statement. These are discussed in turn in the combined results and discussion section. Direct quotes are from interviews with committee members, unless otherwise specified, and are written in italic.

### Literature review

A review of the literature reporting on the impact of public involvement on research was undertaken in 2009 [10]. The findings have been supported by three subsequent reviews [11–13]. INVOLVE, (an organisation funded by the National Institute for Health Research to support public involvement in NHS, public health and social care research) has continued to review the published evidence of impact and produced an online bibliography [14]. We drew on the evidence from these reviews, and discuss some of the findings in conjunction with the results from the interviews.

### Developing written patient statements

In 2012, TwoCan Associates (a small company specialising in promoting and supporting public involvement in research) were commissioned to develop two written patient statements for the NHS NSCT to inform the work of the Advisory Group for National Specialised Services (AGNSS), one relating to atypical hemolytic-uremic syndrome (aHUS) and one relating to transthyretin familial amyloid polyneuropathy (TTR-FAP). AGNSS was a committee that considered services and drugs for very rare conditions (conditions which affect less than 500 people in the UK). NICE took over this assessment of highly specialised technologies from 1 April 2013. We have drawn on this experience to inform our conclusions.

## Results and discussion

### General overview

In general terms, committee members reported that written patient statements offer a very different perspective when evaluating the clinical and economic data, enabling members to answer the question ‘What does this mean for patients?’

*When the micro-technicalities take over the discussion, it enables the chair or one of the committee to say ‘What does the actual patient think about this? Is this really an issue?’* (Committee member 3)

The written patient statement thus provides a counterbalance to the committee’s professionalised views. This is similar to the experience of clinical researchers who describe the input from involved patients as a ‘reality-check’[15].

*The perspective from a committee room is quite artificial… the value of [a written patient statement] is that it is real. It connects information about people’s lives with the decision being made. You can miss that when you are talking about costs or trial data or pharmacological indications… The risk of not taking this into account is that the committee ends up going off on a tangent…* (Committee member 7)

On occasion, the patients’ views have had a profound impact on decision-making (see the example of the review of insulin-glargine below) when committee members have drawn conclusions based on the clinical and economic data that do not reflect the reality of the patient experience.

*From time to time, what patients have said has been an absolute lightbulb moment, a fantastic insight that you wouldn’t get from anywhere else, but that is occasional.*(Committee member 4)

More specifically, the written patient statement has provided patients’ and also carers’ perspectives on:

- The nature of the condition i.e., explaining what the clinical description means in real life
- The clinical benefits and side-effects of the new technology i.e., what the ‘numbers’ mean in real life
- The wider impacts of the new technology i.e., the impacts that matter to patients and carers that may not have been captured through the clinical and economic research
- The relevance of the research findings i.e., whether the clinical trials have assessed what matters to patients and carers

These issues will now be discussed in turn, followed by consideration of the barriers to making use of the information from patients.

### The nature of the health condition under review

The written patient statement often provides valuable background information that enables the committee to put the evidence into a real-life context. It includes details of:

- the natural history of the condition-when it starts in life, how it changes over time, and whether it is life-threatening
- the range of ways that patients are affected and the different ways that different groups of patients may be affected - in terms of impacts on health
- the ways in which the lives of patients, family members, partners and carers are affected-the physical, psychological, emotional, social and financial impacts
- how the condition affects patients’ and carers’ quality of life
- the positive and negative impacts of treatments that are currently available

Patients and carers are able to provide unique insights based on their direct experience of a condition, which are distinct from other expert views, as one committee member described:

*Even clinicians who are familiar with the condition-do they really know what the lives of people are like? Even for them the [written patient statement] can be an eye-opener.* (Committee member 1)

By providing this information in their own words, the patients also aid the committee’s understanding of clinical descriptions of a health condition. For example, a clinical description of amyloidosis might state that the condition results in ‘rapidly-progressing peripheral neuropathy’. What this means in real-life becomes much clearer when described by a patient as follows:

*I’ve lost sensation in my fingers which means I can’t use a keyboard. I’ve found I can use an iPad, but am reluctant to spend all that money when I can’t be sure I’ll still be able to use it in six months’ time.* (Quote from a patient interviewed for a written patient statement for AGNSS)

### The clinical benefits and side-effects of the new technology

Committee members reported that the information from patients helps to translate the quantitative, clinical data into real-life experience. This is useful in assessing whether the reported benefits are significant and valued by patients, and helps with ‘making sense of the numbers’.

*It gives some substance to what would otherwise just be quality of life numbers*. (Committee member 5)

In another example, where the clinical impact of a new technology was described as an increase in visual acuity, based on scores from reading a Snellen chart, the patients explained that this level of improvement meant, for example, that they were then able to see well enough to drive, and therefore able to return to work.

It can also be important to look at these benefits from the patients’ perspective, because patients may place much greater value on small improvements, than someone in good health ‘looking in from the outside’. Making judgements about the value of small improvements was recognised by some committee members as a challenging aspect of the appraisal process.

*Without the patient’s voice, it’s easier to be a little bit more dismissive if you’re looking at clinical data… rather than hearing what effect it had on the individual patient.* (Committee member 2)

Committee members also valued hearing the patients’ views on the side-effects of new technologies. This had sometimes challenged the committee’s assumptions about their impact.

*There was a patient who said ‘I’m taking this drug, but I’ve had to stop it for a couple of days, because it gives me such bad diarrhoea that I wouldn’t have been able to come to this meeting…’ It was that insight - on the page they [the manufacturers] say ‘Side-effects-X% of people get gastrointestinal problems’, but actually that illustration was wow, this is much more important than it appears on a list of adverse effects.* (Committee member 4)

### The wider impacts of the new technology

The written patient statement provides information about impacts that are not included in the clinical and economic data, in particular, impacts beyond clinical outcomes, and impacts on people other than the patient.

*If, for the carer, the treatment means for example they can sleep all night-that would be an incredible step forward-but that wouldn’t have been measured.* (Committee member 1)

In another example, patients provided a different perspective on the clinical data, which profoundly changed the Committee’s thinking:

*We were considering insulin-glargine and the evidence showed that using conventional insulin and insulin-glargine had the same effects on HbA1c [a biomarker for diabetes control] but the glargine cost loads more, but what the committee heard from the patients was that if you have any tendency towards hypoglycaemic events, which can happen with standard insulin, then you literally went to bed every night scared you weren’t going to wake up as a consequence of having a hypo. So people wouldn’t take their insulin and their base level of HbA1c was much higher. So the committee asked for work to be done to survey patients to see how common this behavioural response was, and what impact the higher HbA1c levels would have on survival. Glargine did not result in hypos so had less behavioural impacts-you could take it and run yourself at the appropriate HbA1c level. With the additional evidence, the committee was convinced that a proportion of patients would respond better that way.* (Committee member 5)

### The relevance of the research findings

For committee members, it is important to understand whether a new technology addresses what patients consider to be a priority symptom. This is unlikely to be revealed in trial data. Importantly, this information can only come from the patients themselves, as clinicians may hold different opinions.

*On occasions there’s a discrepancy between the clinicians and the patients… what clinicians think is important and what patients think is important is not always the same. Sometimes what clinicians think is terribly important, patients will say ‘I’ve learned to live with that’.* (Committee member 4)

Such discrepancies have also been reported in clinical research, where patients’ priorities for research on their condition have been shown to be different to those of clinicians and those of researchers [15–19].

Patients are also in the unique position of being able to comment on whether the outcome measures used in clinical trials actually measure what matters to them. Several studies have identified discrepancies between the treatment outcomes that patients prefer, and those that clinicians *believe* patients prefer [20–25]. An appraisal of a new treatment for psoriasis revealed such a distinction. In their statement, the patients commented that clinical benefit is often measured in terms of a % reduction in the areas of skin affected by psoriasis, but what matters to them is whether the psoriasis is visible or present on their joints, as this has much more of an impact on their daily lives. As one Committee member commented:

*What it can do [the written patient statement] is make you realise that what’s been measured may not get to the heart of what’s going on.* (Committee member 5)

### Barriers to making use of the information from patients

Although committee members were aware of the potential value of written patient statements as illustrated by their examples above, all but one expressed concerns about incorporating the patient perspective into the decision-making process-on the basis that the patients’ information does not appear to constitute ‘robust evidence’ (see Table 1). There is a tendency to try to weigh-up the patients’ views *against* the clinical and economic data, which proves difficult.

**Table 1:** The differences between ‘evidence’ and ‘insights’ from patients

The nature of ‘evidence’	The nature of experiential knowledge
• Data obtained through systematic enquiry	• Knowledge gained through experience - insight
• Objective	• Subjective
• Rational	• Emotional
• Written in scientific and technical language	• Written in the patients’ own words
• Mostly quantitative - statistics	• Mostly qualitative - stories
Apples	Pears

*We need objective evidence presented to us, that we can be reassured will accurately tell us what is going on for these patients… part of the problem here [with the written patient statement] is a concern about quality.* (Committee member 7)

*It’s really important to hear the stories from real patients, but it’s not good enough as evidence*. (Committee member 8)

*The challenge is that there’s an ill person who will be pulling on your heartstrings possibly, and that is set against this more objective evidence*. (Committee member 9)

However, there was also some recognition that the written patient statement offers a different kind of knowledge or insight, one that can be valued in its own right.

*It’s not about robustness-this is information about how half a dozen people feel. We need to see them [the written patient statement and the clinical and economic data] as apples and pears. Part of the issue about judgement at this [national] level, is to be able to make judgements between apples and pears-not just reducing everything to apples.* (Committee member 1)

## Conclusion

In undertaking this review of how written patient statements are used by committee members during technology appraisal, we have increased our understanding of how this information adds value in practice. We conclude that it provides a very different form of knowledge to experimental data and functions in a very different way to ‘evidence’.

### How written patient statements are used in practice

**Fig. 1:**
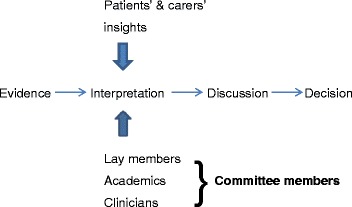
Where do the patients’ insights add value to the decision-making process?

The written patient statement adds value to the committee’s decision-making process by providing the real-life context to the use of the technology and by ‘bringing the numbers to life’. Perhaps most importantly it acts to challenge assumptions that others make based on the clinical and economic data alone. We therefore conclude that the role of the written patient statement is to aid the Committee’s interpretation and understanding of the ‘evidence’. The Committee members have very different backgrounds, and approach this task in different ways. However, they all have one thing in common-none has direct experience of the condition under review. The written patient statement provides this missing perspective as shown in Fig. 1. Only patients are able to draw on their direct experience to evaluate the clinical research evidence.

The information from patients does not therefore need to function as ‘evidence’ in the same way as the clinical and economic data. It does not to sit alongside this evidence. Nor should it be weighed up *against* this evidence. It is better understood as an interpretive tool to aid Committee members in their evaluation and deliberation of the existing clinical and economic data-it enables them to consider this evidence ‘in a different light’.

### The written patient statement provides experiential knowledge or insight

‘Evidence’ within the context of evidence-based medicine takes a particular form - experimental, objective and often quantitative data. The information obtained from patients is very different in nature (see Table 1). Rather than data, it is knowledge gained through experience i.e., insight or wisdom. It is therefore subjective, emotional and often written in the patients’ own words (anecdotal). When judged by the standards required of ‘evidence’ the written patient statement tends to fare poorly, which as one Committee member described is like ‘comparing apples and pears’.

However, in the context of functioning as an aid to understanding and interpreting the clinical and economic data, the experiential knowledge of patients works extremely well. In particular, patients’ ‘stories’ have the power to communicate and challenge Committee members’ assumptions. For example, in the review of insulin-glargine (described above) a patient saying ‘I don’t take my insulin at night in case I don’t wake up in the morning’ brought the Committee’s attention to an important gap in the clinical and economic data, in a way that a professionalised statement about ‘non-adherence to treatment’ might not have done. For this reason, we conclude that if we aim to increase the ‘robustness’ of the written patient statement, in effect turning it into a piece of qualitative research, we would be in danger of reducing its value. If professionals without direct experience of a condition first interpret what patients say, and then report the findings using technical ‘jargon’, this professionalised account may distort the original meaning and it may lose its power to challenge and influence. For this reason, we suggest that the process of developing the written patient statement might be better understood as a consultation to obtain patients’ and carers’ perspectives (informed by their direct experience) on the significance and relevance of existing clinical data, rather than a piece of research to produce additional ‘evidence’ of the patients’ experience.

It is of note, that although we asked interviewees about their use of the *written* patient statement from patient organisations, when reflecting on examples of where the patients’ contributions had made a profound impact on the decision-making process, some committee members referred back to verbal comments made by individual expert patients attending an appraisal meeting. This may be because experiential knowledge or insights have greater resonance and impact when heard directly from the individual concerned. i.e., when heard ‘from the horse’s mouth’. This observation merits further exploration, as there are important implications for the selection and role of patient experts. The findings from this project may also be of relevance to the written summaries produced by these experts.

### What are the implications for practice?

To date, the value of the written patient statement has been understood as providing an understanding of patients’ experience of a health condition and their use of current treatments. Our analysis shows it has the potential to do much more than that. It also provides insights into the significance and relevance of the clinical and economic data. We conclude that a written patient statement needs to broaden from describing a condition and considering the wider social and psychological issues, to also include patients’ and carers’ views on the existing clinical data. This means asking patients and carers questions such as:

- Does the new technology address a symptom/issue that’s a priority?
- What do the reported clinical benefits mean in real-life for patients and carers? What are the wider impacts?
- How ‘tolerable’ are the side-effects?
- Has this clinical trial measured what matters to patients and carers?

Furthermore, there has been little clarity to date on how the written patient statement should be used within the context of health technology appraisal. Although the results of this project are limited by the fact that we only interviewed a small number of appraisal committee members, we conclude that all stakeholders involved may benefit from a greater awareness of the way in which patients’ and carers’ experiential knowledge can add value to the interpretation stage of the decision-making process. This may help appraisal committee members to make better use of the information, by countering any misperceptions that this information should take the form of research evidence. It may also provide greater clarity to patient organisations as to which aspects of the patients’ experience and insights are most valuable for the committee to hear about and which are likely to usefully inform the committee’s deliberations. Further research is required to develop clear guidance on the development and use of written patient statements, both for appraisal committee members and patient organisations.

## Abbreviations

AGNSS: Advisory Group for National Specialised Services
aHUS: atypical hemolytic-uremic syndrome
NHS: National Health Service
NICE: National Institute for Health and Care Excellence
NSCT: National Specialised Commissioning Team
TTR-FAP: transthyretin familial amyloid polyneuropathy
